# Post-Concussion Syndrome Following Blast Injury: A Cross-Sectional Study of Beirut Blast Casualties

**DOI:** 10.5811/westjem.21131

**Published:** 2025-05-18

**Authors:** Hind Anan, Moustafa Al Hariri, Eveline Hitti, Firas Kobeissy, Afif Mufarrij

**Affiliations:** * American University of Beirut Medical Center, Department of Emergency Medicine, Beirut, Lebanon; † Qatar University College of Medicine, Tamayuz Simulation Center, QU Health Sector, Doha, Qatar; ‡ American University of Beirut, Department of Biochemistry and Molecular Genetics, Beirut, Lebanon; § Morehouse School of Medicine, Department of Neurobiology, Atlanta, Georgia

## Abstract

**Introduction:**

The massive 2020 blast in Beirut, Lebanon, caused by improperly stored ammonium nitrate, was one of the most powerful non-nuclear explosions in history, Following the blast, head injuries emerged as a predominant presentation to the emergency department (ED). Blast-induced head injuries can lead to mild traumatic brain injuries (mTBI) mediated via primary blast overpressure without direct head trauma. The recovery process from mTBIs can be prolonged and affected by several factors. If symptoms persist for more than three months, patients should be evaluated for post-concussion syndrome (PCS). While clinical blast-injury studies have focused on repetitive blast exposure, this study evaluates a cohort exposed to a single blast. We hypothesized that a single blast exposure is sufficient to induce PCS symptoms similar to those exposed to repetitive blasts.

**Methods:**

This cross-sectional study explores PCS in patients presenting to the ED of a tertiary-care center following the Beirut blast. Patients were identified through medical charts, contacted by phone, and consented to participate at least three months post-blast (beginning in November 2020). We used the Rivermead Post-Concussion Questionnaire (RPQ) to assess for PCS. We analyzed the association of PCS with patients and injury characteristics.

**Results:**

Of 370 patients presenting to the ED, 145 (58.5%) completed the study questionnaire. Mean age was 39.8 ± 15.4 years, and 40% were females. Head trauma (46.9%) was the most common presentation. A total of 112 patients (77.2%) met the criteria for PCS, with a median RPQ score of 25 (interquartile range 18.75). After adjusting for injury types and distance from the blast, younger patients (adjusted odds ratio [aOR] 0.972, 95% confidence interval [CI] 0.947–0.998) and females (aOR 2.836, 95% CI 1.114–7.220) were more likely to suffer from PCS.

**Conclusion:**

Our study revealed a remarkably high prevalence of PCS among survivors of the Beirut blast, with younger individuals and females disproportionately affected. This highlights the need for age- and sex-specific rehabilitation and support programs. However, the study was limited by incomplete patients records and contact information, leading to the exclusion of a significant number of patients who initially presented to the ED. Ultimately, this study underscores the crucial role of robust public health preparedness and specialized care pathways against future large-scale catastrophes. Further assessment, including neurobiomarker evaluation, will be conducted on these survivors.

## INTRODUCTION

On August 4, 2020, 2.7 kilotons of improperly stored ammonium nitrate accidentally detonated in the Port of Beirut, Lebanon, resulting in one of the most devastating urban explosions in history.^1^ Equivalent to a 3.3 magnitude earthquake, the blast generated a supersonic positive pressure wave followed by a protracted vacuum, with a seismic shockwave traveling up to 250 kilometers (km) and causing widespread destruction as fast as 10 km away.^1^,^2^ This catastrophic event claimed over 200 lives and injured around 7,000 individuals.^3^

A prominent injury type observed in the aftermath was head trauma, with variations in prevalence across major hospitals in Beirut. One center reported 34% of patients suffering head injuries,^4^ while another found 46.9% among those presenting within three days of the blast^5^ and 20.1% overall.^6^ Multicenter studies further confirmed this range, with head injuries accounting for 26.4–40.3% of casualties.^7^,^8^ Among these head injuries, a significant subset emerged: blast-induced traumatic brain injuries (bTBI) as a form of mild traumatic brain injuries (mTBI). Defined as “an alteration in brain function, or other evidence of brain pathology, caused by an external force,”^9^ bTBIs form a distinct and complex category within the broader spectrum of TBIs. Classification primarily hinges on the cause of injury, categorized as primary, secondary, and tertiary. Secondary and tertiary bTBIs result from projectile contact or physical displacement forces, respectively.^10^

However, the mechanism of primary bTBI, caused by the direct impact of the high-frequency pressure wave, remains enigmatic.^10^ Animal studies propose several hypotheses, including shearing of brain tissue due to acceleration, direct skull absorption of the pressure leading to brain damage, and transmission of the pressure wave through hollow organs, then transmitted via veins and arteries and potentially breaching the blood-brain barrier. 10,11 Although primary blast injuries to other organs has been long recognized in the International Classification of Diseases (ICD), it was not until October 2022 that the ICD 10^th^ modification added “Primary blast injury of brain, not elsewhere classified.”^12^

Similar to other forms of TBIs, bTBI can be classified into three categories—mild, moderate, or severe—based on the severity of the injury.13 Clinically, mTBI, often used interchangeably with concussion,14 presents with brief loss of consciousness (less than 30 minutes), post-traumatic amnesia (less than 24 hours), and a Glasgow Coma Score (GCS) of 13–15.15 Symptoms commonly include headaches, dizziness, disorientation, poor concentration, emotional lability, and irritability.14 While most patients recover within two weeks,16 persistent symptoms exceeding three months define post-concussion syndrome (PCS).17 Recovery times are influenced by several factors such as age,18–20 sex,20–22 distance from the blast,23 and presence of associated injuries.24 Prolonged recovery poses significant burdens on individuals and society, with PCS patients experiencing reduced quality of life, increased number of hospitalizations, and lower work return rates.25 Given the Beirut blast’s magnitude and the high prevalence of head injuries, understanding the long-term impact on survivors, particularly the development of PCS, becomes crucial. In this study we aimed to assess the prevalence of PCS among patients who presented to the emergency department (ED) at one of the largest tertiary-care centers following the explosion.

**Population Health Research Capsule**

What do we already know about this issue?

*The 2020 Beirut blast resulted in widespread injuries, with head trauma being prominent. Some patients experienced prolonged recovery and post-concussion syndrome (PCS).*

What was the research question?

*What was the prevalence of PCS among patients presenting to the ED after the Beirut blast?*

What was the major inding of the study?

*Of 145 patients, 77.2% met PCS criteria. Younger (aOR 0.97, p=0.04) and female (aOR 2.84, p=0.03) patients were more affected.*

How does this improve population health?

*The study emphasizes the need for age- and sex-specific rehabilitation for blast survivors, which would help improve recovery, public health preparedness, and disaster response.*

## METHODS

### Patient Identification and Data Collection

This cross-sectional study is reported in accordance with Strengthening the Reporting of Observational Study in Epidemiology (STROBE) guidelines and approved by the institutional review board (Protocol ID: BIO-2020-0357).

Given that concussion can occur without direct head trauma and can be caused solely by the blast pressure, all patients who presented to the ED of the tertiary-care center on the day of the explosion and the following three days (August 4–7, 2020) were identified using electronic health records. Those patients who could be reached via a phone number were included in the study. We used medical charts to collect patient demographics and injury characteristics.

Starting in November 2020, three months after the blast, patients were contacted by phone and orally consented to participate in the study. We collected variables pertaining to their presentation and exact location on the day of the blast. Patients were also asked about the severity of the concussion symptoms they experienced in the prior 24 hours compared to pre-blast. We used Google Earth to calculate the patient’s distance from the blast location.

### Primary Outcome

To assess the primary outcome, PCS, we used the Rivermead Post-Concussion Questionnaire (RPQ), a validated questionnaire with good test-retest and inter-rater reliability.^26^ The RPQ inquires about 16 concussion symptoms experienced in the prior 24 hours as compared to the pre-injury period. Participants rate each symptom on a Likert scale of 0 (not experienced at all), 1 (no more of a problem than pre-injury), 2 (a mild problem), 3 (a moderate problem), and 4 (a severe problem). Scores of “1” were considered as “0” since they were symptoms present before the injury,^26^,^27^ resulting in a total score range of 0–64. We defined PCS as the presence of ≥3 symptoms on the RPQ (score of ≥2 on each symptom).^28^ We then categorized the severity of PCS into minimal (0–12), mild (13–24), moderate (25–32), and severe (≥33) based on the levels of self-reported symptoms.^29^

### Statistical Analysis

We performed statistical analysis using SPSS Statistics for Windows, v28.0 (IBM Corp., Armonk, NY). Two-sided *p* < 0.05 was considered to be statistically significant. We present categorical variables as percentages and frequencies, while continuous variables are expressed as means ± standard deviation or median with interquartile range (IQR). We evaluated the differences between PCS groups using Pearson chi-square or Fisher exact test for categorical variables and the Student *t*-test for continuous variables. We constructed a multivariable logistic regression model to determine independent predictors of PCS. Variables were included in the model if they were clinically relevant or found to be significant on bivariate analysis.

## RESULTS

**Figure 1:**
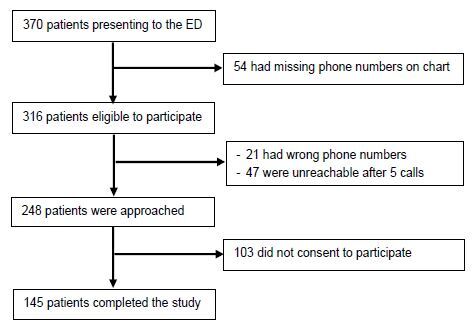
Flow chart describing the identification and enrollment of patients presenting to the ED* following the Beirut, Lebanon, explosion in August 2020. *ED*, emergency department.

A total of 370 patients presented to the ED between August 4–7, 2020, of whom 248 (67.0%) were eligible to participate in the study. Among them, 103 patients refused to consent, while 145 patients (58.5%) completed the study questionnaire and were included in the final analysis (Figure 1).

The mean age of participating patients was 39.8 ± 15.4 years, with 58 (40%) being females. Fifty-six (38.6%) of the injured patients were within a one km radius of the blast epicenter. Head trauma (46.9%) and alteration in mental status (45.5%) were the two most common presentations, while 42 (29.0%) patients did not present with any neurological complaint. The most frequently injured body part was the upper extremity (19.9%), followed by the head/face (19.1%) (Table 1).

**Figure 2:**
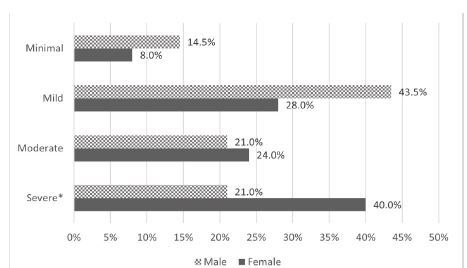
The severity of PCS categorized by sex among patients presenting to the ED following the Beirut blast. *p<0.05. *ED*, emergency department; *PCS*, post-concussion syndrome.

The majority of patients (77.2%) met the criteria for PCS. Among them, 13 (11.6%) experienced minimal symptoms, 41 (36.6%) had mild symptoms, 25 (22.3%) had a moderate presentation, and 33 (29.5%) suffered from severe symptoms three months after the blast. On bivariate analysis, sex (female) was found to be the only variable associated with having PCS (*p*=0.04) (Table 1). Additionally, being female was significantly associated with having severe symptoms (Figure 2).

Patients with PCS mostly suffered from fatigue (81.3%), noise sensitivity (78.6%), restlessness (77.7%), sleep disturbances (74.1%), and feeling depressed (73.2%). Light sensitivity (17.0%) was the least reported symptom. On the other hand, those with no PCS (22.8%) complained mostly of headaches (21.2%), fatigue (18.2%), and frustration (15.2%) (Table 2).

The multivariate logistic regression assessing the association between PCS and participants’ characteristics showed that age and sex were independent predictors of PCS. Younger people (adjusted odds ratio [aOR] 0.972, *p*=0.04, 95% confidence interval [CI] 0.947–0.998) were at higher risk of having PCS. Similarly, females were more likely to meet the criteria for PCS (aOR 2.884, *p*=0.03, 95% CI 1.133–7.340) (Table 3).

## DISCUSSION

Traumatic brain injury poses a socioeconomic burden on both the affected patients and society, for the patients’ decreased quality of life, more frequent hospitalizations, and lower rates of returning to work.^25^ Our study showed a high prevalence of PCS following the Port of Beirut blast. More than three-quarters of the study participants met the criteria for PCS three months later. This prevalence is considerably higher than reported in other disaster settings, where rates typically range from 10–40%.^30^–^33^ This could be attributed to the scale of the blast that detonated in a densely populated area of the Lebanese capital and to the type of injuries resulting from combined primary, secondary, and tertiary mechanisms and requiring urgent medical presentations to the ED. Notably, females were significantly more likely to experience PCS overall and had a higher proportion of severe cases. These findings highlight this specific blast event’s profound and lasting impact on its survivors, particularly women.

Unlike most studies on PCS resulting from accidents or falls, we examined the aftermath of a singular, non-natural event. The Beirut blast’s unique combination of a high-pressure wave injury,^2^ physical debris impact, and psychological trauma presents a distinct set of challenges and potential long-term consequences.^2^,^5^,^34^ While many studies in the literature have extensively investigated bTBIs and their prognosis in military settings, research on civilian bTBIs remains scarce. Of interest, our study participants were all civilian victims who sustained physical injuries beyond head trauma, which will offer valuable and unique insights into the broader public health implications of such catastrophic events. Additionally, existing military bTBI research focused typically on the development of PCS, altered mental health outcomes, and neuropsychological symptoms resulting from repetitive low-level or high-level blast exposures.^35^ In fact, it was shown that the severity of PCS cumulatively increases as a function of a number of blast exposures.^35^ In contrast, in our study the Beirut blast constitutes a single, high-level blast exposure that was found to be associated with altered neuropsychological changes represented by PCS.

Our finding that younger individuals were at higher risk of developing PCS aligns with prior research on TBI. A study by Ponsford et al found that younger mTBI patients were more associated with higher reporting of PCS symptoms on follow-up.^36^ Additionally, Mearse et al reported a trend for an association between younger age and PCS, although this trend did not reach statistical significance.^31^ This trend aligns with the developing brain’s heightened vulnerability to injury and suggests that younger individuals may require targeted interventions and support following blast events. Nevertheless, some reports highlighted that older age is an independent risk factor for unfavorable outcomes at follow-up for PCS patients.^19^,^20^,^37^ Moreover, Garza et al showed that with every year of age, there was a 2.6% increase in the odds of an unfavorable outcome.^18^ Further research with larger and more diverse samples is needed to clarify the relationship between age and PCS risk in the context of blast injuries.

The increased risk of PCS among women in our study aligns with some existing research, indicating a potential sex-based vulnerability to TBI complications. Multiple studies reported that females were more likely to experience PCS compared to males.^36^,^38^,^39^ Additionally, females were more likely to report higher symptom severity in total score and all somatosensory, vestibular, affective, and cognitive symptom clusters than males.^20^–^22^,^40^ This disparity may be attributed to hormonal differences, pre-existing conditions, or variations in pain processing between the sexes.^36^,^38^ Further research is needed to understand the biological and social factors contributing to this sex-based disparity in PCS outcomes and develop sex-specific management strategies.

While previous studies have linked closer proximity to the blast with increased blast-related injury severity,^23^,^41^ our study did not find a significant association between distance from the blast and PCS diagnosis. It is possible that this is due to the fact that the majority of our cohort was within 3 km of the blast center.^5^ Our study enrolled patients seeking medical attention at the ED, potentially excluding individuals with milder injuries who resided further from the blast site and preferred to present to other medical centers since our institution was among the centers that sustained damage from the blast.^42^ Additionally, we calculated the radius distance from the center of the blast not taking into consideration barriers that reflect blast wave. Future investigations with larger and geographically diverse samples are needed to clarify the relationship between distance and PCS occurrence in blast events.

Finally, PCS remains a subject of ongoing debate in the scientific community. While the ICD-10 still recognizes PCS as a distinct clinical entity,^43^ it has been removed from the *Diagnostic and Statistical Manual of Mental Disorders*, 5^th^ edition, and reclassified under “neurocognitive disorder due to traumatic brain injury.”^44^ Some argue that PCS symptoms do not meet the criteria for a “syndrome”^45^ and instead use the term “persistent post-concussive symptoms” (PPCS).^46^ Recent efforts have been aimed at redefining PCS and PPCS as persisting symptoms after concussion, emphasizing that not all symptoms occurring after a concussion are necessarily caused by the concussion.^47^ This controversy underscores the complex nature of PCS and highlights the need for multidisciplinary approaches to its study and treatment. Continued research is essential to clarify its diagnostic criteria and improve patient outcomes.

## LIMITATIONS

Our study was conducted in an ED in the setting of a catastrophic, unexpected event. This led to a substantial number of patients with missing or incomplete medical charts and contact information. As a result, a significant number of the patients who initially presented to our ED were not included in the study. However, data on symptoms and location at time of injury were comprehensively confirmed on follow-up phone calls. Furthermore, the single-center nature of the study, located near the blast site, may limit generalizability. Most of our cohort was within 3 km of the blast, potentially excluding those with milder injuries who sought care elsewhere.

It is also important to note that symptoms of PCS are not encountered exclusively in patients with TBI and may overlap with other disorders.^22^,^30^ This study was further limited by the scientific controversy surrounding PCS^47^ as a distinct clinical entity. While our findings depict the prevalence of PCS symptoms following the blast, they cannot conclusively attribute PCS to bTBI. Additionally, we were unable to specifically classify patients as having primary, secondary, or tertiary bTBIs, as they may have suffered from one or a combination of any of these subtypes for determing the severity of the bTBI.

## CONCLUSION

This study highlights the significant prevalence and enduring impact of post-concussive syndrome symptoms following the catastrophic explosion in the Port of Beirut, particularly among women and younger individuals. These findings underscore the need for tailored rehabilitation and support programs for blast survivors, considering age- and sex-specific vulnerabilities. Additionally, our investigation emphasizes the importance of studying civilian blast events to inform comprehensive public health preparedness plans and disaster response strategies. Investing in robust mental health resources and specialized care pathways for blast-related traumatic brain injury should be a priority in building resilience against future large-scale catastrophes.

## Figures and Tables

**Table 1:** Demographic and injury characteristics of patients affected by injuries following the Beirut blast.

Variable		Total (145)	No PCS (33)	PCS (112)	P-value
Age (years, mean ± SD)		39.8 ± 15.4	44.1 ± 16.5	38.5 ± 14.9	0.07
Female		58 (40.0%)	8 (24.2%)	50 (44.6%)	0.04
Comorbidity*		24 (18.5%)	5 (15.6%)	19 (19.4%)	0.63
Neurological presentation					
Head trauma		68 (46.9%)	17 (51.5%)	51 (45.5%)	0.55
Loss of consciousness		29 (20.0%)	6 (18.2%)	23 (20.5%)	0.77
Pre- or post-blast amnesia		37 (25.5%)	10 (30.3%)	27 (24.1%)	0.47
Alteration in mental status		66 (45.5%)	15 (45.5%)	51 (45.5%)	0.99
Any other neurological deficit		26 (17.9%)	6 (18.2%)	20 (17.9%)	0.97
None		42 (29.0%)	10 (30.3%)	32 (28.6%)	0.85
Body part injured					
Head/Face		27 (19.1%)	7 (21.9%)	20 (18.3%)	0.66
Spine		2 (1.4%)	0 (0.0%)	2 (1.8%)	1.00
Shoulder/Arm		28 (19.9%)	5 (15.6%)	23 (21.1%)	0.49
Hip/Leg		21 (14.9%)	2 (6.3%)	19 (17.4%)	0.16
Ear		3 (2.1%)	1 (3.1%)	2 (1.8%)	0.54
Abdomen/Pelvis		1 (0.7%)	0 (0.0%)	1 (0.9%)	1.00
Skin		17 (12.1%)	4 (12.5%)	13 (11.9%)	0.93
Location at the time of the blast					
Distance from the blast (km, mean ± SD)		1.7 ± 1.2	1.7 ± 1.2	1.7 ± 1.2	0.89
Zones	< 1 km	56 (38.6%)	11 (33.3%)	45 (40.2%)	
	1 km –3 km	36 (24.8%)	10 (30.3%)	26 (23.2%)	0.66
	> 3 km	53 (36.6%)	12 (36.4%)	41 (36.6%)	

* Patients were considered to have comorbidity if they had at least one of the following: chronic obstructive pulmonary disease; hypertension; diabetes; coronary artery disease; hyperlipidemia; or history of neurological disease.

*PCS*, post-concussion syndrome; *RPQ*, Rivermead post-concussion questionnaire; *IQR, i*nterquartile range.

**Table 2:** Rivermead post-concussion syndrome questionnaire symptoms experienced by patients at least three months after the Beirut blast.

RPQ Symptoms		Total (145)	No PCS (33)	PCS (112)
Somatic symptoms	Headaches	71 (49.0%)	7 (21.2%)	64 (57.1%)
	Dizziness	55 (37.9%)	2 (6.1%)	53 (47.3%)
	Nausea/vomiting	37 (25.5%)	1 (3.0%)	36 (32.1%)
	Fatigue	97 (66.9%)	6 (18.2%)	91 (81.3%)
	Noise sensitivity	90 (62.1%)	2 (6.1%)	88 (78.6%)
	Light sensitivity	19 (13.1%)	0 (0.0%)	19 (17.0%)
	Blurred vision	64 (44.1%)	1 (3.0%)	63 (56.3%)
	Double vision	64 (44.1%)	4 (12.1%)	60 (53.6%)
	Sleep disturbance	87 (60.0%)	4 (12.1%)	83 (74.1%)
Cognitive symptoms	Forgetfulness	54 (37.2%)	0 (0.0%)	54 (48.2%)
	Poor concentration	35 (24.1%)	0 (0.0%)	35 (31.3%)
	Longer to think	72 (49.7%)	3 (9.1%)	69 (61.6%)
Emotional symptoms	Being irritable	40 (27.6%)	0 (0.0%)	40 (35.7%)
	Feeling depressed	86 (59.3%)	4 (12.1%)	82 (73.2%)
	Feeling frustrated	82 (56.6%)	5 (15.2%)	77 (68.8%)
	Restlessness	89 (61.4%)	2 (6.1%)	87 (77.7%)
RPQ score (median (IQR))		20 (21)	2.0 (6.0)	25 (18.75)

*PCS*, post-concussion syndrome; *RPQ*, Rivermead post-concussion questionnaire; *IQR*, interquartile range.

**Table 3:** Logistic regression analysis assessing the association between the characteristics of patients and injury types with the likelihood of developing post-concussion syndrome following the Beirut blast.

Variable	aOR	p-value	95% CI
Age (Continuous)	0.972	0.04	(0.947–0.998)
Gender (Ref: Male)	2.836	0.03	(1.114–7.220)
Injury other than head (Ref: No)	1.104	0.82	(0.468–2.604)
Head trauma (Ref: No)	0.769	0.53	(0.339–1.744)
Distance from the blast (Continuous)	1.000	0.89	(1.000–1.000)

*aOR*, adjusted odds ratio; *95% CI*, confidence interval; *Ref*, reference.
